# Diagnostic accuracy of different CMR techniques in the evaluation of ischemic cardiomyopathy

**DOI:** 10.1186/1532-429X-15-S1-E71

**Published:** 2013-01-30

**Authors:** Eugene Won, Angela Tong, Ruth P Lim, Tatyana Danilov, Leon Axel, Monvadi B Srichai

**Affiliations:** 1 Medicine, Radiology, NYU School of Medicine, New York, NY, USA; 2 Medicine, Beth Israel Hospital, New York, NY, USA; 3 Radiology, Austin Health, Melbourne, VIC, Australia; 4 New York Medical College, Valhalla, NY, USA

## Background

In patients newly diagnosed with heart failure, coronary angiography is the current gold standard in differentiating underlying coronary artery disease and non-ischemic cardiomyopathy. However, CMR is emerging as an attractive alternative that can provide substantially more diagnostic information with its multiple sequences, while also demonstrating a superior safety profile. In this study, we evaluated the diagnostic ability of late gadolinium enhancement (LGE), cine, and first pass perfusion (FPP) in diagnosing ischemic cardiomyopathy compared to coronary angiography (CA).

## Methods

Retrospective chart review identified 109 consecutive patients (68% male, average age 58 (range 23-84 years), average ejection fraction 30% (range 10-50%) referred for CMR evaluation of new onset cardiomyopathy with CA performed within 2 weeks of CMR. Both imaging tests were performed within two months of clinical heart failure diagnosis. Patients were examined with cine, FPP, and LGE sequences as part of the CMR protocol. Ischemic cardiomyopathy was defined based on the standardized criteria proposed by Felker *et al.* - stenosis of 70% or greater in the left main vessel or proximal LAD or at least two epicardial coronary arteries as identified on CA. Cine images were evaluated for regional and global wall motion abnormalities (hypokinesia/dyskinesia). FPP images were evaluated for presence of regional hypoperfusion. LGE images were evaluated for subendocardial and/or transmural enhancement consistent with an ischemic pattern in a coronary artery distribution. These sequences were then analyzed for their predictive value in the diagnosis of ischemic cardiomyopathy.

## Results

CA identified 66 patients (60.6%) with ischemic cardiomyopathy. Diagnostic accuracy, sensitivity, specificity, PPV, NPV and ROC analysis of individual and combinations of techniques for diagnosis of ischemic cardiomyopathy are presented in Table 1. Using information from all three techniques resulted in near perfect sensitivity at the expense of specificity. McNemar test comparing diagnostic accuracies of tests showed that LGE-CMR alone was significantly better than the other two sequences alone.

**Table 1:** Diagnostic accuracy, sensitivity, specificity, positive predictive value, and negative predictive value for the combinations of CMR techniques in diagnosing ischemic cardiomyopathy compared to CA.

Combination of CMR sequences	Sensitivity	Specificity	Positive predictive value	Negative predictive value	diagnostic accuracy	ROC
**CINE**	61%	60%	70%	50%	61%	0.61
**FPP**	83%	49%	72%	65%	67%	0.64
**LGE**	89%	63%	79%	79%	79%	0.76
**CINE, FPP**	94%	35%	69%	79%	71%	0.64
**LGE, CINE**	94%	44%	72%	83%	74%	0.69
**LGE, FPP**	97%	42%	72%	90%	75%	0.69
**CINE, FPP, LGE**	97%	30%	68%	89%	71%	0.64

CINE = regional wall motion abnormality on cine, FPP = regional hypoperfusion on resting first pass perfusion, LGE = ischemic pattern on late gadolinium enhancement

## Conclusions

Ischemic pattern of LGE alone demonstrates the best diagnostic performance for diagnosis of ischemic cardiomyopathy compared to other individual and combination CMR techniques. A combination of techniques may be needed to improve the diagnostic sensitivity and negative predictive value in order to ensure identification of patients who may benefit from revascularization.

## Funding

None.

